# Supplementary material for: Outcomes of hospitalized patients with COVID-19 according to level of frailty
Source: PeerJ. 2021 Apr 13;9:e11260. doi: 10.7717/peerj.11260 (PMC8051355; doi:10.7717/peerj.11260)
Supplement: Supplemental Information 1 — This codebook converts numbers to their respective factors. [file peerj-09-11260-s001.pdf]

CODE BOOK

ICU. Intensive Care Unit

0 = Not
1 = Yes

CFS res

CFS. Score. Clinical Frailty Score

1 = Frail
2= Pre-Frail
3 = Robust.

CFS. Classification. Clinical Frailty Score

3 = Frail
4= Pre-Frail
5 = Robust

Age

Value in years.

Sex

1 = Man
2 = Woman

Health Care (Provider)

1 = Not
2 = Yes

Postal Code

Number of Postal Code

Overcrowding

0= Not
1 = Yes

Living Positive Covid

0 = Not
1= Yes

Country Born

Country Born

Functional Sate

1 = Independent
2 = Semi-Dependent
3= Dependent

Nosocomial Transmission

0 = Not
1= Yes

Center of Admission

HULP: Hospital La Paz
CIII: Hospital Carlos III
CANT: Hospital Cantoblanco
Days Admission

Floor

Floor of Admission.

Readmision

0 = Not
1= Yes

Cardiovascular Diseases

0 = Not
1= Yes

Hypertension

0 = Not
1= Yes

COPD

0 = Not
1 = yes

Oxygen at Home

0=Not
1 = yes

Asthma

0 = Not
1= Yes

Kidney Disease

0=Not
1 = yes

GLOMERULAR FILTERING_30

0=Not
1 = yes

Liver Disease Acuate

0=Not
1 = yes

Liver Disease Mild

0=Not
1 = yes

Neoplasm_ Malignant

0=Not
1 = yes

AIDS

0=Not
1 = yes

Obesity

0=Not
1 = yes

Diabetes

0=Not
1 = yes

Diabetes without Complications

0=Not
1 = yes

Rheumatological Diseases

0=Not
1 = yes

Dementia

0=Not
1 = yes

Malnutrition

0=Not
1 = yes

Dyslipemia

0=Not
1 = yes

Mental_Problem_Commun

0=Not
1 = yes

Mental Problem Acuate 1-2

0=Not
1 = yes

Pregnancy

0=Not
1 = yes

Post Pregnancy

0=Not
1 = yes

Smoker

0=Not
1 = yes

Smoker Habits

0=Not
1 = yes

Tobacco_packs_year

Value

Psoriasis

0=Not
1 = yes

Dermatitis

0=Not
1 = yes

Hydradenitis

0=Not
1 = yes

Trips

0=Not
1 = yes

Trips in Spain

0=Not
1 = yes

Risks Factors

0=Not
1 = Yes

Special Risk Factors

Description

Charson Comorbidity index

Value in the scale

Date Begging Sintoms

Date

Date Arriving Emergency

Date

Date Discharge

Date

Date Hospitalization

Date

Oxigenotherapy

0=Not
1 = Yes

Prone

0=Not
1 = Yes

Peep

0=Not
1 = Yes

IPAP

0=Not
1 = Yes

Respiratory Frequency

Value

Need to admission Intensive Care

0=Not
1 = Yes

Arrangement Beds

0=Not
1 = Yes

Readmision Intensive Care

0=Not
1 = Yes

Exitus

0=Not
1 = Yes

Date Exitus

Date

Disease Not Complicate

0=Not
1 = Yes

Mild Pneumonia

0=Not
1 = Yes

Acuate Pneumonia

0=Not
1 = Yes

Respiratory Distress

0=Not
1 = Yes

Sepsis

0=Not
1 = Yes

Septic Shock

0=Not
1 = Yes

Fever

0=Not
1 = Yes

Myalgia

0=Not
1 = Yes

Discomfort

0=Not
1 = Yes

Myalgia

0=Not
1 = Yes

Rhinorrhea

0=Not
1 = Yes

Dysgeusia

0=Not
1 = Yes

Anosmia

0=Not
1 = Yes

Cough

0=Not
1 = Yes

Productive Cough

0=Not
1 = Yes

Odynofagia

0=Not
1 = Yes

Pain Chest

0=Not
1 = Yes

Pain Rib

0=Not
1 = Yes

Hemoptysis

0=Not
1 = Yes

Dyspnea

0=Not
1 = Yes

Pain Abdominal

0=Not
1 = Yes

Diarrhea

0=Not
1 = Yes

Sickness

0=Not
1 = Yes

Vomits

0=Not
1 = Yes

High Level Awareness

0=Not
1 = Yes

Behaviors

0=Not
1 = Yes

Convulsion

0=Not
1 = Yes

Infection Admission

0=Not
1 = Yes

Microorganism Laboratory

0=Not
1 = Yes

Microorganism Name

Name

Pneumonia Bacteria

0=Not
1 = Yes

ARDS

0=Not
1 = Yes

Pneumothorax

0=Not
1 = Yes

Disease Pleural

0=Not
1 = Yes

Meningitis

0=Not
1 = Yes

Convulsion (Status)

0=Not
1 = Yes

<u>Stroke</u>

0=Not
1 = Yes

<u>Heart Failure</u>

0=Not
1 = Yes

<u>Myocarditis</u>

0=Not
1 = Yes

<u>Pericarditis</u>

0=Not
1 = Yes

<u>Endocarditis</u>

0=Not
1 = Yes

<u>Arrythmia</u>

0=Not
1 = Yes

<u>Cardiac Arrest</u>

0=Not
1 = Yes

<u>Cardiac Arrest by VF</u>

0=Not
1 = Yes

<u>Alteration Coagulation</u>

0=Not
1 = Yes

Anemia Subsidiary

0=Not
1 = Yes

Rhabdomyolysis

0=Not
1 = Yes

Digestive Bleeding

0=Not
1 = Yes

Pancreatitis

0=Not
1 = Yes

Liver Failure

0=Not
1 = Yes

Accuate Confusional Syndrome

0=Not
1 = Yes

Psyquiatrics Complications

0=Not
1 = Yes

Drugs Reactions Mild

0=Not
1 = Yes

Drugs Reactions Acuate

0=Not
1 = Yes

Drug Reaction

0=Not
1 = Yes

Number Confirmed PCR

Number of PCR

Date PCR Negative

Date

Evolution Complete

0=Not
1 = Mild
2 = Yes

Scale Cursg 65

Valor Corresponding in the scale.

Scale Fine 65

Valor Corresponding in the scale.

Scale QSofa 65

Valor Corresponding in the scale.

Scale SOFA 65

Valor Corresponding in the scale.

PSI

Scale Cursg 65

Valor Corresponding in the scale.

Diseases Neurological

0=Not
1 = Yes

Diseases Cardiological

0=Not
1 = Yes

Diseases Hematological

0=Not
1 = Yes

Diseases Hematological

0=Not
1 = Yes

Diseases Digestive

0=Not
1 = Yes

Diseases Neuro-Psychiatric

0=Not
1 = Yes

Compilations Infections

0=Not
1 = Yes

Inflammatory Diseases

0=Not
1 = Yes
